# Risk Analysis as Regulatory Science: Toward The Establishment of Standards

**DOI:** 10.1093/rpd/ncw211

**Published:** 2016-09-22

**Authors:** Michio Murakami

**Affiliations:** Department of Health Risk Communication, Fukushima Medical University School of Medicine, Fukushima City, Japan

## Abstract

Understanding how to establish standards is essential for risk communication and also provides perspectives for further study. In this paper, the concept of risk analysis as regulatory science for the establishment of standards is demonstrated through examples of standards for evacuation and provisional regulation values in foods and drinking water. Moreover, academic needs for further studies related to standards are extracted. The concepts of the traditional ‘Standard I’, which has a paternalistic orientation, and ‘Standard II’, established through stakeholder consensus, are then systemized by introducing the current status of the new standards-related movement that developed after the Fukushima nuclear power plant accident, and the perspectives of the standards are discussed. Preparation of standards on the basis of stakeholder consensus through intensive risk dialogue before a potential nuclear power plant accident is suggested to be a promising approach to ensure a safe society and enhance subjective well-being.

## INTRODUCTION

After the Great East Japan Earthquake on 11 March 2011 and the subsequent nuclear power plant accident at Fukushima, on 17 March 2011 the Japanese government announced provisional regulation values for radioactive materials in drinking water and foods to restrict the distribution of contaminated foods through markets. On 11 March 2011 the government also ordered a full evacuation of residents living within 2 km of the Fukushima Daiichi nuclear power plant, and on 12 March 2011 it expanded the evacuation zone to a 20-km radius from the Fukushima Daiichi nuclear power plant and a 10-km radius from the Fukushima Daini nuclear power plant. On 22 April 2011 it again ordered the evacuation, within 1 month, of residents with an additional effective dose of 20 mSv/y, and on 26 August 2011 it targeted these areas to achieve an additional effective dose of 1 mSv/y as a long-term goal through decontamination in addition to physical and environmental decay. The public has become conscious of the issue of radioactive materials in drinking water and foods and of the safety of additional effective doses exceeding these regulation values in habitats, and there have been discussions about whether the regulation values are too high or too low.

Government officials and experts explained through the mass media that the public were safe and did not need to worry, because the detected levels were lower than the regulation values and the regulation values were established with a large safety margin. At a press conference on 19 March 2011, Yukio Edano, chief cabinet secretary at the time, explained the provisional regulation values for radioactive materials in drinking water and foods; he said that these values were estimated with consideration of the health effects of ingestion over a lifetime on the basis of recommendations from the International Commission on Radiological Protection (ICRP)^(1)^ (although this explanation was incorrect, as described later in this paper). In response to these explanations, some members of the public thought that they were still not safe despite values being less than the regulation ones; others thought that the regulation values were not low enough, or that the government or experts were not to be trusted. Through questionnaires conducted in September 2011, Hommerich reported that 70.3% of residents in the prefectures of Fukushima, Iwate and Miyagi (in the Tohoku region) and 75.6% of residents in the prefectures of Tokyo and Kanagawa (in the Kanto region) did not trust government institutions, and that residents’ subjective well-being (happiness) was significantly negatively associated with distrust of government and anxiety about radioactive contamination of foods^(2)^. Because the decrease in subjective well-being in Fukushima and neighboring prefectures after the nuclear power plant accident was also reported to be significantly large (equivalent to 240% of annual income)^(3)^, the roles of trust and anxiety, as well as physical health, are important. Obviously, risk communication in relation to the provisional regulation values or standards was not successful.

Although there has been much discussion of the regulation values themselves, it has rarely been explained how these regulation values were estimated and how safety was defined and achieved. William Thomas Sedgwick (1855–1921) a pioneer of public health in the USA, taught his students that ‘standards are devices to save lazy minds the trouble of thinking’^(4)^. Not only the public but also government officers or even experts, often regard standards as authority; however, to achieve safety in society and communicate risk among stakeholders, an understanding of the concept of safety and the evidence and protocols associated with standards is essential. Murakami *et al*. explored how several standards in various fields (including food safety, the environment, radiological protection and traffic and explosives accidents) were established; they compared the essence of standards among these fields in terms of risk communication with the public or experts^(5, 6)^. In this paper, first, the concept of risk analysis as regulatory science for safety management is demonstrated. Examples of standards for evacuation and provisional regulation values in foods and drinking water are given and the evidence, protocols and academic needs for further studies related to standards are summarized. Finally, the perspectives of standards are discussed on the basis of the concept of Standard I and Standard II, in light of the new standard-related movement that has arisen since the nuclear power plant accident in Japan.

## RISK ANALYSIS AS REGULATORY SCIENCE FOR SAFETY MANAGEMENT

### Safety and risk analysis

Safety is defined as ‘freedom from risk which is not tolerable’ by the third edition of the International Organization for Standardization (ISO)/International Electrotechnical Commission (IEC) Guide 51^(7)^. (Safety is defined as ‘freedom from unacceptable risk’ in the second edition^(8)^). It is not true that safety is objective and reassurance is subjective. For individuals, safety is the combined concept of objective risk assessment and subjective risk perception, and for society safety is a concept composed of risk assessed in a reproducible manner and social consensus related to the level of risk that is not tolerable.

Historically, the concept of risk assessment was born in the latter part of the 17th century. After Pascal and Fermat founded the concept of ‘mathematical probability’^(9)^ in about 1660, the concept of risk was described in the Port Royal ‘Logic’, which followed the thoughts of Pascal: i.e. ‘many people who are excessively terrified when they hear thunder … if it is only the danger of death that fills them with their extraordinary fear, it is easy to show that this is unreasonable … Fear of harm ought to be proportional not merely to the gravity of the harm, but also the probability of the event …’ This concept is similar to the definition of risk in the ISO/IEC Guide 51^(7)^, namely a ‘combination of the probability of occurrence of harm and the severity of that harm’. In the same period as Pascal, Graunt^(9, 10)^ stated that ‘whereas many persons live in great fear and apprehension of some of the more formidable and notorious diseases following; I shall only set down how many died of each: that the respective numbers, being compared with the total 229 250 [the mortality over 20 y], those persons may the better understand the hazard they are in’. This concept is similar to risk comparison, although Graunt did not use the term ‘risk’. Clearly, these two historical books described people's perception of death and their insights into reasonable decision-making. Since these achievements, risk analysis comprising three components (i.e. risk assessment, risk management and risk communication) has been developed as a useful art to support decision-making, including regulatory actions (e.g. establishment of safety standards).

Because safety is achieved through social consensus as well as risk assessment, safety can be judged by using not normal pure science but, instead, regulatory science. Regulatory science is useful for filling the gaps between pure scientific knowledge and decision-making, including in regulatory actions^(11)^. Although the concept of regulatory science is similar to that of trans-science^(12)^, regulatory science places great importance on an outcome-based (or solution-based) approach such as the setting of regulations. Specifically, regulatory science enables evaluation through prediction, assumption and estimation based on scientific knowledge and can therefore respond to social needs. On the other hand, regulatory science works by meeting the demand for regulatory action to advance academic sciences. The US Food and Drug Administration and European Chemicals Agency have now started to adopt the concept of regulatory science for safety management^(13, 14)^. Risk analysis is conducted within the framework of regulatory science and allows us to provide information and to update scientific knowledge for decision-making in the face of uncertainties.

### Protocols for filling the gaps between pure science and establishment of standards

As an example of the establishment of standards on the basis of pure scientific knowledge, linear non-threshold (LNT) theory has been adopted for risk management in both radiological protection and environmental science. This concept was first clarified in the ICRP Publication 1, adopted in 1958^(15)^ in the face of increased concern about low-dose exposure in fields other than medicine (e.g. nuclear power plants and nuclear bomb testing) and epidemiological findings regarding an increase in the incidence of leukemia in radiologists. Here, the concept that the incidence of leukemia was proportional to the accumulated dose was adopted: ‘The most conservative approach would be to assume that there is no threshold and no recovery … and the incidence might be proportional to the accumulated dose’. Namely, this assumption emphasizes ‘how to protect or manage radiological risk’ rather than ‘how to predict the actual probability of cancer incidence’; therefore, LNT theory works as a tool for protection and risk management. Similar approaches were adopted in the environmental standards for chemical carcinogens in water, air and foods. Here, risk is used as an ‘indicator’ for regulation- or decision-making.

Risk analysis under these assumptions has two advantages. First, this concept provides information for making a decision related to risk management. In particular, safety standards were established on the basis of risk analysis. Second, it enables us to compare risks among various factors or to evaluate risk trade-offs. Because individual or social decisions always include risk trade-offs in general, and these trade-offs are also likely to emerge after the disaster (e.g. the risk of having a motor vehicle accident vs. suffering an airplane crash after the terrorist attacks in 2001 in the US^(16)^, or the risk to the elderly from nursing-home evacuation vs. the radiation risk from not evacuating after the nuclear power plant accident in 2011 in Japan^(17)^), evaluation of risk trade-offs is essential to minimize overall risk in society.

For safety management, ‘acceptable risk’ or ‘tolerable risk’ also needs to be defined. The lowest reference level in the effective dose band for emergency exposure situations (20 mSv/y), which was used as the evacuation criterion in Japan and originated from constraints set by the ICRP for occupational exposure^(18)^, was determined mainly by using the acceptable risk level (10^–3^/y) in the UK Royal Society's study group report as a reference^(19)^. In that report, the risk number chosen was referenced to the annual risk of assassination of the President of the US (2%/y); the risk from dangerous situations such as voluntary sports and professional stunting (3–6 × 10^–3^/y); the risk of death from all causes in males aged between 1 and 20 y (<10^–3^/y); the risk of a fatal accident in industries considered dangerous (i.e. quarries, mines, railways and the construction industry) (1–3 × 10^–4^/y); and the risk of accidents in the manufacturing industry (3 × 10^–5^/y). The acceptable risk level was explained as follows: ‘The imposition of a continuing annual risk of death to the individual of 10^–2^ seems unacceptable. At 10^–3^ it may not be totally unacceptable if the individual knows of the situation, enjoys some commensurate benefit, and everything reasonable has been done to reduce the risk’.

Note that this number was clearly established from a paternalistic perspective and justified from the balance between freedom and unacceptable risk. In other words, this standard was established under the concept that the regulator does not allow members of the public the freedom to enjoy benefits if they come with large risks, rather than the concept that the regulator should protect the public from risk. This concept leads to the difficult question: ‘Is the freedom or the willingness to return to a home town contaminated with radiation accepted if three premises (knowledge of the situation, benefit by staying at the home town, and reasonable risk reduction) are met?’ Because radiation is perceived with the most dread among various risks and risk perception differs among individuals^(20)^, some people emphasize avoidance of risk and others prefer to benefit by staying at the home town. Society should respect both value systems. Another issue is that the social community in a municipality is comprised not only of individuals but also of a given number of groups. As mentioned in detail later, evacuation zone cancellations based on much lower doses have been determined from stakeholder consensus in the case of the nuclear power plant accident in Fukushima.

### Need to establish standards as perspectives for further studies: an example of provisional regulation values for foods and drinking water

The reputation of regulatory science is useful for highlighting the need to establish standards and works to advance academic sciences associated with this need. As an example, the lessons learned from provisional regulation values for foods and drinking water are explained in this section. After the nuclear accident in Japan, provisional regulation values were established mainly according to control index levels set by the Nuclear Safety Commission of Japan^(21)^, with the expectation of ^131^I levels of 100 Bq/kg in milk and other dairy products for infants. This value (100 Bq/kg) originated from the guideline levels set by the Codex Alimentarius Commission^(22)^. Historically, food safety regulation has aspects of government intervention on the free market. The control index level set by the Nuclear Safety Commission of Japan was set for intervention in times of emergency and was estimated in consideration of the balance between benefit (risk reduction) and cost, as follows:

$$DIL_{kj}=\frac{ILD/G}{F\times W_{kj}\times \sum_{i}S_{ij}\times f_{i}\times \{1-\exp(-\lambda_{i}\times t)\}/\lambda_{i}}$$

where DIL is the control index level (Bq/kg); *k* is the food category group (^131^I: three groups; sum of ^134^Cs and ^137^Cs: five groups); *j* is the age group [infants (<1 y old), child (5 y old) and adult]; ILD is the intervention level of the dose [^131^I: two-thirds of the additional thyroid equivalent dose of 50 mSv (= ~2 mSv/y of additional effective dose) (the other one-third is reserved for other foods); sum of ^134^Cs and ^137^Cs: 5 mSv/y of additional effective dose); *G* is the number of food category groups; *F* is a dilution factor (^131^I: 1; sum of ^134^Cs and ^137^Cs: 0.5); *W* is the daily consumption rates of drinking water and foods (kg/d); *S* is the committed dose coefficient (mSv/Bq); *t* is the period of ingestion (365 d); *f* is the composition of each radionuclide in relation to the representative radionuclide [^131^I is regarded as representative of the sum of all radioiodine and ^132^Te; ^134^Cs and ^137^Cs are regarded as representative of the sum of all radiocesium and radiostrontium (^137^Cs: ^90^Sr = 1:0.1)]; and *λ* is the physical decay coefficient (d^-1^).

Control index levels were estimated for the three age groups and minimum concentrations established as index levels. Members of the public were assumed to ingest contaminated foods for 1 y, starting just after the emergency, although Yukio Edano, chief cabinet secretary at the time, explained that people were assumed to ingest contaminated foods over a lifetime. Importantly, the concentrations of radionuclides in foods and drinking water were assumed to decrease according to radionuclide physical decay. It was not assumed that the public consumed foods in which radioiodine concentrations were constant. Note that this assumption gives large differences in the estimated numbers: under the assumption that the radioiodine concentration was constant, the calculated control index levels would be approximately one-thirtieth of those estimated under the assumption of decrease^(5)^.

In this estimation protocol, the dilution factor used can be a driving factor for the actual doses received by the general public after the nuclear power plant accident. The dilution factor set by the Nuclear Safety Commission of Japan is very conservative (no dilution for ^131^I and 50% dilution for radiocesium), despite the low food self-sufficiency rates in Japan (54% for major grains; 39% on the basis of total energy^(23)^). As a result, the average doses due to ingestion of ^131^I, ^134^Cs and ^137^Cs were very limited (e.g. 0.062 mSv/y for adults in Fukushima City as the additional effective dose—much lower than the intervention level)^(24)^. Since the dilution factor has a large safety margin, the dose received by the public could be reduced.

Setting a dilution factor is important in determining regulation values. The provisional regulation values for radiocesium (including contributions from radiostrontium) in general foods (excluding drinking water and milk and other dairy products) were set at 500 Bq/kg on the basis of a 50% dilution factor and a 5-mSv/y additional effective dose as an intervention level. Before the accident in Fukushima, the European Union (EU) set the regulation value for radiocesium in general foods as 1250 Bq/kg on the basis of a 10% dilution factor and a 1-mSv/y additional effective dose as the intervention level, whereas the US chose 1200 Bq/kg on the basis of a 30% dilution factor and a 5-mSv intervention level^(25)^. The regulation values in Japan were much stricter than those in the EU and USA, despite the use of 5 mSv/y as the intervention level. In fact, using a 1% dilution factor and 1 mSv as the intervention level yields the same regulation values as using a 100% dilution factor and 100 mSv as the intervention level. Setting the dilution factor should therefore be carefully considered, as setting the intervention level done. Otherwise, the dilution factor can act as an adjustment factor in setting arbitrary regulation values. Further studies are warranted to improve our understanding of dilution factors.

## PERSPECTIVES FOR STANDARDS: STANDARD I AND STANDARD II

Since the nuclear power plant accident at Fukushima, other approaches have been used to establish standards. First, evacuation zones have been canceled by stakeholder consensus; this especially reflects the willingness to listen to citizens who are concerned about radiation exposure. There are three requirements for the cancellation of an evacuation zone, namely

1. the additional effective dose is <20 mSv/y;
2. essential infrastructure and services for daily life have almost returned to normal, and sufficient decontamination has been done (especially in environments for children); and
3. there have been sufficient discussions among prefecture, municipality and inhabitants^(26)^.

However, in practice, rather than being taken as 20 mSv/y, the requirement regarding the additional effective dose has been determined from discussions among stakeholders. For example, an additional effective dose was much lower (1 to 2 mSv/y) when the evacuation zones were canceled in Kawauchi Village in October 2014^(27)^; this reflected the anxiety over radiation exposure.

New, stricter limits for foods were established in April 2012, although even the provisional regulation values resulted in economic damage, which was much larger than the benefit estimated from the willingness to pay to avoid risks^(28)^. The new limit was established by using a 1-mSv/y intervention level in accordance with the Codex Alimentarius Commission (with a 50% dilution factor), and the concept of ‘as low as reasonably achievable (ALARA)’, in order to enhance safety and reassurance^(29)^, especially by considering consumer concerns. This is seemingly in contrast to the decision in Norway after the Chernobyl accident, whereby the government raised the regulation values for radiocesium in reindeer meat^(30)^ with full consideration of the country's unique dietary culture; nevertheless, the Japanese and Norwegian approaches are very similar in that both governments placed emphasis on stakeholders. Interestingly, a local limit for food distribution (20 Bq/kg) was established within a community in Kashiwa City, Chiba Prefecture (adjacent to Tokyo)—a known ‘hot spot’—reflecting the consensus achieved at a meeting among consumers, producers and experts^(31)^. Yasumasa Igarashi, a chief of the secretariat in the meeting, noted the special meaning of the fact that the local limit was determined through consensus among all stakeholders, rather than being simply a calculated value. This local rule allows consumers to be satisfied and producers to manage their crops with pride to achieve the target. These decisions are in accordance with the recommendation of ICRP Publication 111 in 2009^(32)^: ‘For management of the radiological quality of foodstuffs in a country with a contaminated territory, relevant stakeholders (authorities, farmers’ unions, food industry, food distribution, consumer non-governmental organizations, etc.) and representatives of the general population should be involved in deciding whether individual preferences of the consumers should outweigh the need to maintain agricultural production, rehabilitation of rural areas, and a decent living for the affected local community’. This social discussion about radiation exposure has parallels to that about genetically modified foods in the 1990s. The UK government's Select Committee on Science and Technology^(33)^ gained important insights into this discussion: ‘They are not about safety as such, but about much larger questions of what kind of a world we want to live in’. Namely, here, members of the public do not exclusively discuss the risk itself but expect to advance society in the direction they believe to be correct. This movement works to restructure society, thus enabling the public to increase their subjective well-being. In this case, standards are devices used to realize the world people want to live in.

The kind of decision-making and established standards mentioned above (defined as ‘Standard II’) are clearly different from the traditional, paternalism-oriented standard (‘Standard I’); they were named after the concepts of ‘System I and System II’ in psychology^(34)^ or ‘Safety I and Safety II’ in resilience engineering^(35)^. Table 1 compares the concepts of Standard I and Standard II. Standard I, which was established by governments under the concept of paternalism, is applied on a national or global scale and enables reasonable risk reduction. For various risk factors, including radiation exposure, this standard can allow us to balance the risk trade-offs or to achieve the best cost–benefit under the limitation of resources and finances. This standard is also useful for ensuring minimum safety under the condition that risk is regarded as acceptable. Objective risk assessment plays a key role in the procedure of risk analysis used to establish the standard. In contrast, Standard II, which was established by stakeholder consensus, can be applied at a local scale in general (and possibly also at a national scale). It becomes more important in the case of hazards, of which society is particularly aware (e.g. nuclear power plant accidents and genetically modified foods), along with social penetration of consumer rights, as proposed by John F. Kennedy. This standard emphasizes realization of the ‘kind of world we want to live in’. This allows stakeholders to be satisfied with, or proud of, the risk management system, which is supported by the finding that risk acceptance can be governed by trust^(36)^. Rather than the size of the risk itself, subjective well-being (happiness), which has recently been adopted in the field of economics to evaluate utility^(37)^, can be a key indicator for assessing the status of society. In addition to traditional risk assessment, risk communication (as the meaning of ‘dialogue’) or risk governance on the basis of stakeholder engagement plays a key role in establishing this type of standard.

**Table 1.** Concepts of Standard I and Standard II.

	Standard I	Standard II
Main player for establishment of standards	Government or regulators	Stakeholders
Orientation of decision-making process	Paternalism	Consensus
Objective	Risk reduction	Realization of ‘the kind of world we want to live in’
Principal goal	Assurance of reasonable safety (at minimum)	Enhancement of satisfaction and pride
Area of application	National or global	Local or national
Target for assessment	Objective risk (mortality rate etc.)	Subjective/social values (happiness etc.)
Key role/input for risk management	Risk assessment	Risk dialogue/ risk governance

The nuclear power plant accident and radiation risks have become social issues, probably reflecting greater risk perception among members of the public^(20)^. The traditional Standard I is always needed to achieve reasonable safety in accordance with the balance between risk and cost; however, the lessons learned from the accident at Fukushima highlight the importance of the role of Standard II.

Although the Japanese government responded rapidly, regulating foods and drinking water and deciding on evacuation zones, thus leading to significant risk reductions^(24, 38)^, the failure of risk communication about standards has disrupted public well-being. Both, preparation of standards and sufficient risk communication before a potential nuclear power plant accident, are important to reduce risk and to well-being. Although Standard II has still generally been applied since the nuclear power plant accident, it would have been a promising approach to apply Standard II before the accident in order to establish standards through intensive discussion toward social consensus. This may warrant the expansion of further academic fields. Greater understanding of the types of standards that allow the public to enjoy high levels of subjective well-being would enable risk to be managed well and would eventually achieve a safe society for all to want to live in.

## FUNDING

This study was supported by a CREST grant from the Japan Science and Technology Agency, JPSP KAKENHI Grant Number JP15H01774, and the national "Health Fund for Children and Adults Affected by the Nuclear Incident". The findings and conclusions of this article are solely the responsibility of the authors and do not represent the official views of Fukushima Prefecture government.
